# Effect of percutaneous minimally invasive pedicle screw internal fixation in the treatment of thoracolumbar vertebral fractures and its impact on quality of life

**DOI:** 10.12669/pjms.38.1.4329

**Published:** 2022

**Authors:** Pengfa Tu, Chong-chao Yan, Jian-xue Hao, Shuo Cao, Chenyang Jiang

**Affiliations:** 1 Pengfa Tu, Department of Orthopaedics, Baoding No.1 Hospital, Baoding, Hebei, 071000, P.R. China; 2 Chong-chao Yan, Department of Orthopaedics, Baoding No.1 Hospital, Baoding, Hebei, 071000, P.R. China; 3 Jian-xue Hao, Department of Orthopaedics, Baoding No.1 Hospital, Baoding, Hebei, 071000, P.R. China; 4 Shuo Cao, Department of Orthopaedics, Baoding First Central Hospital, Baoding, Hebei, 071000, P.R. China; 5 Chenyang Jiang, Department of Orthopaedics, Baoding No.1 Hospital, Baoding, Hebei, 071000, P.R. China

**Keywords:** Percutaneous minimally invasive, Pedicle screw fixation, Thoracolumbar segment, Vertebral fracture

## Abstract

**Objectives:**

To investigate and analyze the effect of percutaneous minimally invasive pedicle screw internal fixation in the treatment of thoracolumbar vertebral fractures and its impact on quality of life.

**Methods:**

Fifty patients with thoracolumbar vertebral fracture admitted to our hospital from January 2015 to December 2018 were selected and divided into two groups according to different treatment regimens. The observation group was treated with minimally invasive percutaneous pedicle screw internal fixation, while the control group was treated with traditional posterior approach open pedicle screw internal fixation. The surgery time, incision length, intraoperative blood loss, postoperative drainage, hospitalization time, ambulation time, fracture healing time and postoperative VAS scores were compared between the two groups. In addition, the cobb angle, the sagittal plane index, and the anterior vertebral height were compared between the two groups before and after surgery, as were the Oswestry Disability Index (ODI) at 1d, 3 months, and 6 months postoperatively.

**Results:**

The surgery time, incision length, postoperative pain level, postoperative drainage and intraoperative blood loss of the observation group were less than those of the control group (P<0.05). The postoperative Cobb angle of the two groups decreased, the sagittal plane index as well as the anterior vertebral height increased (P<0.05). The Oswestry index of the observation group was better than that of the control group at one day and three months postoperatively, with a statistical significance between the two groups (P<0.05). The complication rate of the observation group was significantly lower than that of the control group (P<0.05).

**Conclusion:**

Percutaneous minimally invasive pedicle screw internal fixation is safer than the traditional open pedicle screw internal fixation, and it is more worthy of clinical promotion.

## INTRODUCTION

Vertebral fracture, as a common disease in the orthopedic clinic, is mainly characterized by thoracolumbar spine (T11 - L2) fractures.^1^ Spinal fractures without reliable fixation or delayed treatment may result in nerve damage and even paraplegia.^2^ Currently, pedicle screw internal fixation is used clinically to treat thoracolumbar spine fractures. With this therapy scheme, the nail-rod system is used to restore and fix the fracture site, so that the vertebral body returns to the normal sequence relationship and height level, which is conducive to improving vertebral stability and relieving nerve compression.^3^,^4^

It has been shown in some studies that traditional open surgery will produce many unfavorable effects, such as increased risk of vascular and nerve damage, severe tissue dissection, obvious postoperative pain, large intraoperative bleeding, prolonged hospital stay, and reduced patient quality of life.^5^ With the development of orthopedic internal fixation instruments and medical imaging, as well as the improvement of surgical strategy, percutaneous minimally invasive pedicle screw internal fixation has achieved significant curative effect in the treatment of thoracolumbar spine fractures, which can significantly reduce the pain of patients, shorten the hospitalization time, reduce intraoperative bleeding, and improve the quality of life of patients.^6^

This study was carried out with its main purpose of investigating the effect of minimally invasive percutaneous pedicle screw fixation in the treatment of thoracolumbar vertebral fractures and its impact on the quality of life. The specific details are reported as follows.

## METHODS

### Ethical approval:

The study was approved by the Institutional Ethics Committee of Baoding No.1 Hospital,( Feb. 24^th^ 2021) and written informed consents were obtained from all participants.

### Subjects:

In this retrospective study was used in this study. A total of 50 patients with thoracolumbar vertebral fracture admitted to our hospital from January 2015 to December 2018 were selected and divided into two groups according to different treatment regimens: the observation group (n=25) and the control group (n=25). The observation group ranged in age from 19 to 67 years old, including 13 males and 12 females, with an average age of (45.10±6.40) years. While the control group ranged in age from 20 to 61 years old, including 15 males and 10 females, with an average age of (42.60±4.20) years. There was no significant difference between the two groups in terms of age, gender, fracture site, fracture types and other data, which was comparable (P>0.05). See Table-I for details. Out of the 50 cases, there were 9 cases injured by falling from a height, 17 cases of traffic accident injury, 9 cases of squeeze and 15 cases of plane fall injury. In terms of fracture grade, these 50 cases could be divided into 9 cases of L1 fracture, 8 cases of L2 fracture, 10 cases of L3 fracture, 11 cases of T-11 fracture, and 12 cases of T-12 fracture. Table-I

**Table I:** Comparison of general information between the two groups.

Group	n	Gender (Male/Female)	Age	Fracture classification (cases)	Site of the fracture (cases)	Fracture reason (cases)

				A1/A2/B2	L1/L2/L3/T11/T12	Falling from a height/traffic accident/squeeze/plane fall injury
Observation group	25	13/12	45.10±6.4	10/10/5	4/4/5/5/7	4/9/5/7
Control group	25	15/10	42.6±4.2	9/12/4	5/4/5/6/5	5/8/4/8
x^2^/t/z	-	0.458	0.870	0.325	0.472	0.413
P	-	>0.05	>0.05	>0.05	>0.05	>0.05

***Note:*** A1: Compression fracture; A2: Split fracture; B2: A fracture with injury to the bony structure behind the spine.

### Inclusion criteria:

• Patients diagnosed with thoracolumbar vertebral fracture by diagnosis and imaging, and classified as A1, A2, and B2 according to the AO classification of spinal fractures
• Patients who received a 6-month follow-up period.

### Exclusion criteria:

• Patients with incomplete general information or who withdrew from the study
• Patients with mental disorder or cognitive impairment
• Patients with open fractures
• Patients with severe dysfunction of heart, liver, kidney, lung and other visceral organs
• Patients with coronary heart disease, diabetes and other basic diseases
• Patients who were assessed as intolerable to surgery
• Patients who received conservative treatment and accompanied by obvious nerve damage.

### Traditional open pedicle screw fixation:

X-ray, CT, MRI and other imaging examinations were utilized to determine the specific location of thoracolumbar vertebral fracture preoperatively. The patients underwent general anesthesia in the prone position. After the completion of preoperative preparation, the surgery was carried out through posterior midline approach with the injured vertebra as the center. A 10-15cm long posterior median longitudinal incision was performed to expose the injured vertebra and the upper and lower vertebrae. Reduction and internal fixation and decompression were performed as appropriate based on the patient’s condition. Four to six fixation screws^7^ were placed on both sides of the injured vertebrae and the upper and lower vertebrae respectively, and the vertebral connecting rods were installed to expand the vertebrae and restore the anterior vertebral height. X-ray fluoroscopy was used to determine whether the reduction reached the ideal state.^8^ Finally, routine suture and indwelling drainage tube were performed.

After the completion of preoperative preparation, the surgery was performed. The patient was placed in the prone position, and general anesthesia was achieved through inhalation and intravenous anesthesia. A C-arm X-ray machine was used to locate the pedicles of the upper and lower vertebral bodies of the injured vertebrae under fluoroscopy, and a transverse incision of 1.8 to 2 cm^9^ deep to the fascia was made on the skin. Under fluoroscopic monitoring, the bone cortex was drilled near the outer cortex edge in the pedicle oval shadow, and the puncture needle was inserted about 1 cm to the posterior edge of the vertebral body. The guide wire was retained, the working cannula was placed one by one, the depth was measured, the suitable pedicle screw was selected, the suitable fixation rod was selected, the fixation rod was placed in the slot, and then the fixation screw was screwed in. Fixation rods were placed on the opposite side in the same manner. The X-rays of the C-arm were changed to the lateral position, and the brace device was placed into both sides at the same time via small incisions at the upper and lower levels. Under fluoroscopy, the brace was opened and reduced to correct the deformity.^10^,^11^ When the reduction reached the ideal state, the screws were fixed, and routine rinsing and suture were performed.

The surgery time, incision length, intraoperative blood loss, postoperative drainage, hospital stay, ambulation time and fracture line healing time were compared between the two groups. The postoperative VAS scores were compared between the two groups. VAS score^8^: unacceptable pain (five points); severe pain (four points); moderate pain (three points); mild pain (two points); no pain (one point). (3) The cobb angle, the sagittal plane index, and the anterior vertebral height were compared between the two groups before and after surgery (4) The Oswestry Disability Index (ODI) was compared between the two groups 1d, 3 months and 6 months after surgery.

### Statistical methods:

SPSS23.0 was used for statistical analysis. The measurement data were expressed as mean ± standard deviation (x̄± s), and compared by the *t* test. The enumeration data were compared using the *χ*^2^ test. P<0.05 indicates a statistically significant difference.

## RESULTS

The surgery time and incision length of the observation group were shorter than those of the control group, and its postoperative drainage and intraoperative blood loss were less than those of the control group, with a statistical significance (P<0.05). The hospitalization time, fracture healing time and ambulation time of the observation group were less than those of the control group, with no statistical significance (P>0.05). Table-II

**Table II:** Comparison of surgery related indexes between the two groups (x̅ ±S).

Group	n	Surgery time (min)	Incision length (cm)	Postoperative drainage(ml)	Intraoperative blood loss(ml)	Hospitalization time(d)	Healing time of fracture line (week)	Ambulation time (week)
Control group	25	66.7±17.4	12.6±3.5	164.5±42.3	268.3±45.7	15.2±1.9	12.4±0.6	6.5±3.4
Observation group	25	44.1±8.6	1.7±0.6	16.4±1.8	18.2±24.6	10.4±1.7	10.2±0.5	2.2±2.4
t value	-	4.093	3.062	555.250	2.651	1.249	1.720	2.006
P value	-	<0.05	<0.05	<0.05	<0.05	>0.05	>0.05	>0.05

The postoperative pain score of the observation group was significantly lower than that of the control group, with a statistically significant comparison between the two groups (P<0.05). Table-III for details. Cobb angle decreased, sagittal plane index and anterior vertebral height increased in two groups postoperatively. The preoperative and postoperative comparisons were statistically significant (P<0.05). Table-IV .

**Table III:** Comparison of postoperative pain scores between the two groups (x̅ ±S).

Group	n	1d after surgery	7d after surgery	14d after surgery	21d after surgery
Control group	25	3.1±0.5	3.0±0.6	2.6±0.4	2.1±0.2
Observation group	25	2.6±0.4	2.2±0.1	1.8±0.4	1.4±0.1
t value	-	1.562	9.32	11.41	14.05
P value	-	<0.05	<0.05	<0.05	<0.05

**Table IV:** Comparison of imaging indexes between two groups of patients with thoracolumbar vertebral fractures (x̅ ±S).

Group	n	Cobb angle(º)		Sagittal index (º)		Anterior vertebral height (%)	

		Before surgery	After surgery	Before surgery	After surgery	Before surgery	After surgery
Control group	25	16.8±6.0	7.3±1.2	21.3±12.9	8.7±2.6	65.8±11.5	87.6±5.00
Observation group	25	15.7±5.7	6.6±1.2	21.2±4.0	8.5±2.2	62.5±10.5	88.7±6.3
t value	-	1.108	1.000	10.400	1.396	1.199	1.587
P value	-	<0.05	<0.05	<0.05	<0.05	<0.05	<0.05

The Oswestry index of the observation group was better than that of the control group at one day and three months after surgery, with a statistical significance between the two groups (P<0.05). However, there was no statistical significance between the two groups at six months after surgery (P>0.05). Table-V.

**Table V:** Comparison of Oswestry indexes between the two groups (x̅ ±S).

Group	n	1d after surgery	3 months after surgery	6 months after surgery
Control group	25	60.03±5.19	14.16±4.25	10.54±1.56
Observation group	25	57.42±6.25	7.25±4.82	11.25±1.24
t value	-	1.23	1.286	1.652
P value	-	<0.05	<0.05	>0.05

The incidence of complications in the control group was 5 (20.00%), including one case of nerve stimulation symptom, two cases of deep vein thrombosis, one case of delayed wound healing due to fat liquefaction, and one case of superficial wound infection; In contrast, the incidence of complications in the observation group was 1 (4.00%), including one case of local pain at pedicle screw placement. The comparison between the two groups was statistically significant (P<0.05).

## DISCUSSION

A variety of adverse effects will be caused by thoracolumbar vertebral fracture on patients, such as vertebra instability, vertebral collapse and deformation, or in severe cases, cauda equina nerve and bone marrow damage, leading to paralysis of patients.^13^ For the purpose of maintaining the stability of the vertebra and restoring the anatomical height, pedicle internal fixation will be used to fix the injured vertebrae on the upper and lower normal vertebrae with screws to help fracture healing.^12^ Traditional open pedicle screw internal fixation is simple in operation and can achieve the effect of reduction and fixation. However, many adverse effects will be caused to patients, such as excessive intraoperative blood loss, large trauma area, easy to cause spinal nerve dorsal branch injury and infection, serious dissection of paraspinal fascia and muscle, which is not conducive to the recovery of patients.^13^,^14^

With the continuous development of medical technology, percutaneous minimally invasive pedicle screw internal fixation has gradually been widely used clinically to treat thoracolumbar vertebral fractures.^15^ It has been shown in some studies that in the comparison of clinical efficacy, percutaneous minimally invasive pedicle screw internal fixation is far superior to traditional open pedicle screw internal fixation^16^ because of its significant advantages, such as small incision, less muscle dissection and loss, the use of C-arm X-ray to locate the fracture, precise reduction, shorter hospital stay and less postoperative pain and other complications caused by soft tissue separation. The results of this study also indicate that the minimally invasive percutaneous pedicle screw fixation requires shorter surgical duration, shorter incision length, shorter hospital stay, and shorter fracture line healing time than the traditional open-cut pedicle screw fixation.

It is of great importance to select the appropriate surgical indications for patients who are about to undergo percutaneous minimally invasive pedicle screw internal fixation,^17^,^18^ such as patients with unstable burst fractures (requiring no space occupying or space occupying in the spinal canal, but less than 1/3 of the space occupying, and without neurological complications), patients with simple compression fractures, and non-surgical treatment patients (chronic bronchitis, mental illness, accompanied by venous thrombosis, etc.)^19^ Only via preoperative scientific evaluation of the prognosis of patients, intraoperative rational utilization of C-arm X-ray machine fluoroscopy, accurate positioning of screw placement point, and favourable reduction of fracture with matching instruments, can the postoperative efficacy be guaranteed.

The results of Zou et al. showed that minimally invasive percutaneous pedicle screw internal fixation was significantly superior to open pedicle screw internal fixation in terms of soft tissue dissection length, intraoperative blood loss, postoperative drainage, and postoperative VAS score at one postoperative day.^20^ This study also found that the postoperative drainage volume and intraoperative blood loss of the observation group were significantly lower than those of the control group, and the postoperative pain score was also significantly lower than that of the control group, which was similar to the results of previous studies. Lin et al.^21^ believed that minimally invasive percutaneous pedicle screw internal fixation had obvious advantages in the treatment of thoracolumbar fracture and had good fracture reduction effect through the evaluation of ODI score, fixed segmental kyphotic Cobb Angle, VAS score at 3 days after surgery, etc. In our study, postoperative Cobb Angle decreased, sagittal plane index increased, and prevertebral height increased in both groups, and the changes in the observation group were more obvious. The Oswestry index of the observation group was better than that of the control group one day and three months after operation.

### Limitations of this study:

The number of cases included in this study was 50, which was limited, so the conclusions drawn may not be very convincing. In this study, percutaneous minimally invasive pedicle screw internal fixation proved to be superior to the open procedure, but further studies are needed to include more patients and set longer follow-up periods to draw more conclusive conclusions.

## CONCLUSION

Both traditional open pedicle screw internal fixation and percutaneous minimally invasive pedicle screw internal fixation have achieved the effect of reduction and fixation. Percutaneous minimally invasive pedicle screw internal fixation is safer than the traditional open pedicle screw internal fixation, and it is more worthy of clinical promotion.

### Authors’ Contributions:

**PT& CCY:** Designed this study and prepared this manuscript.

**JXH & CJ:** Collected and analyzed clinical data, are responsible for the accuracy and completeness of this study.

**SC:** Significantly revised this manuscript.
